# Exploring *Gunnera tinctoria*: From Nutritional and Anti-Tumoral Properties to Phytosome Development Following Structural Arrangement Based on Molecular Docking

**DOI:** 10.3390/molecules26195935

**Published:** 2021-09-30

**Authors:** Faezeh Fathi, Samad N. Ebrahimi, Ana I. G. Valadão, Nelson Andrade, Anabela S. G. Costa, Cláudia Silva, Alireza Fathi, Peyman Salehi, Fátima Martel, Rita C. Alves, Maria Beatriz P. P. Oliveira

**Affiliations:** 1 Department of Phytochemistry, Medicinal Plants and Drugs Research Institute, Shahid Beheshti University, Evin, Tehran 1983969411, Iran; FFathi@ff.up.pt (F.F.); S_ebrahimi@sbu.ac.ir (S.N.E.); P_salehi@sbu.ac.ir (P.S.); 2 REQUIMTE/LAQV, Department of Chemical Sciences, Faculty of Pharmacy, University of Porto, Rua Jorge Viterbo Ferreira No. 280, 4050-313 Porto, Portugal; valadaoana97@gmail.com (A.I.G.V.); nelsonandrade@outlook.com (N.A.); anabelac020@gmail.com (A.S.G.C.); rcalves@ff.up.pt (R.C.A.); 3 Unit of Biochemistry, Department of Biomedicine, Faculty of Medicine of Porto, University of Porto, Al. Prof. Hernâni Monteiro, 4200-319 Porto, Portugal; rakelclaudia@hotmail.com (C.S.); fmartel@med.up.pt (F.M.); 4 Instituto de Investigação e Inovação em Saúde (I3S), University of Porto, R. Alfredo Allen, 208, 4200-135 Porto, Portugal; 5 Sana Technologists Segal Private Company (STM), Ashrafi Esfahani, Tehran 1469963811, Iran; alirezafathi139@gmail.com

**Keywords:** bioactive compounds, phytosome, secondary layer, molecular docking, bilayer arrangement

## Abstract

*Gunnera tinctoria*, an underexplored invasive plant found in Azores, Portugal, was studied regarding its nutritional, antioxidant, and antitumoral properties. Higher antioxidant activity was found in baby leaves, followed by adult leaves and inflorescences. A phenolic fraction of the plant was enriched using adsorbent resin column chromatography (Diaion^TM^ HP20LX, and Relite EXA90). Antitumoral effects were observed with the enriched fractions in breast (MCF-7) and pancreatic (AsPC-1) cancer cell lines, being more pronounced in the latter. To improve protection and membrane absorption rates of phenolic compounds, nano-phytosomes and cholesterol-conjugated phytosomes coated with natural polymers were loaded with the enriched fraction. The particles were characterized, and their physiochemical properties were evaluated and compared. All samples presented anionic charge and nanometer size in relation to the inner layer and micrometer size regarding the external layers. In addition, the molecular arrangement of phenolics within both types of phytosomes were studied for the first time by molecular docking. Polarity and molecular size were key factors on the molecular arrangement of the lipid bilayer. In conclusion, *G. tinctoria* showed to be an interesting source of nutrients and phenolic compounds with anti-tumoral potential. Moreover, phytosome loading with these compounds can increase their stability and bioavailability having in view future applications.

## 1. Introduction

Natural plant sources produce multiplied decisive effects on human health attributed to potent therapeutic properties [1]. *Gunnera tinctoria*, a highly competitive plant named Nalca, belongs to the Gunneracea family [2] and to the subgenus panke (molina) Schindler [3]. It is cultivated in South America [4] being typically found in São Miguel Island, Azores, Portugal, as an invasive plant which is seen as a threat against endemic cultures [5]. However, in native areas, like Chile, *G. tinctoria* has been used as a part of food in salads, sweets, or ice creams [6]. Dysentery, diarrhea, circulatory and urinary disorders, high respiratory tract diseases, and gynecological, and obstetric problems, are some examples where *G. tinctoria* is used in folk medicine [7]. Moreover, antioxidant, dermal anti-inflammatory, and antifungal activities, as well as improvement of the endothelial function are recent therapeutic activities that have been also reported for *G. tinctoria* [8]. Based on this, this plant emerges as a particularly interesting natural source of bioactive compounds.

Regrettably, cancer is a major cause of death in both developed and developing countries. It compasses a group of diseases involving abnormal cell proliferation with potential to spread to other parts of the body. Despite the efforts aiming to improve all treatment methods, early diagnosis and novel and advanced therapeutic interventions are still needed. Besides, plant extracts are mostly rich in bioactive compounds, especially phenolic compounds (PCs), which possess many anticarcinogenic properties, including inhibition of cancer cell proliferation, tumor growth, angiogenesis, metastasis, and inflammation, as well as pro-apoptotic effects. Therefore, it is vital to continue to search for anticancer agents/compounds in plants to find potential therapeutic targets that can be safe and reduce the side effects induced by chemotherapy [9]. 

Moreover, considering the high structural sensitivity and low bioavailability of natural products, especially PCs [10], the search for appropriate natural-based techniques to improve the low bioavailability and stability of such compounds, preventing their decomposition through the digestive system or during storage, is indispensable. Thereupon, encapsulation by natural polymers can provide a natural protection to PCs, improving their bioavailability, bioacessibility, and bioactivity [11]. Moreover, encapsulation of natural products based on lipid carriers similar to those of cell membrane can improve desirable features and cover undesirable properties of natural products, attracting the interest of potential consumers. 

In this work, the nutritional composition of different parts of *Gunnera tinctoria*, namely, root, inflorescence, baby and adult leaf were analyzed in terms of total protein, fat, insoluble and soluble fiber, and available carbohydrates contents. Total phenolics and flavonoids and the antioxidant activity of *G. tinctoria* crude extracts (using DPPH^●^ scavenging and ferric reducing antioxidant power (FRAP) assays) have been also evaluated for the different parts of the plant. Afterwards, a phenolic enriched fraction of *G. tinctoria* was encapsulated based on a lipid layer (nano-phytosome/cholesterol-conjugated phytosome) and coated by binary polymeric layers. The physiochemical properties of the micro/nanoparticles obtained were characterized. Furthermore, the molecular arrangement of the lipid layers were analyzed by membrane generator (MemGen). The molecular interactions between the lipid layer and phenolic compounds were investigated by computational simulation regarding molecular modeling (Docking), which is typically used to create the active binding site of ligand to receptor especially in protein [12,13]. Indeed, this study presents, for the first time, the molecular arrangement of PCs within the phytosome layer by molecular docking. Besides, the variant formation of the polymeric coating layer was estimated schematically throughout a phytosome layer for the first time, as well. 

## 2. Results and Discussion

### 2.1. Nutritional Composition

Table 1 describes the nutritional composition of different parts of *G. tinctoria.* Adult leaves presented significantly higher (*p* < 0.05) contents of total fiber (44 g/100 g dry weight (dw)) and protein (14 g/100 g dw) compared to the other parts of the plant. In terms of insoluble fiber, there were no significant differences (*p* > 0.05) between adult leaves (40 g/100 g dw) and inflorescences (39 g/100 g dw), both being richer than baby leaves (32 g/100 g dw) and roots (10 g/100 g dw). The soluble fiber contents were quite similar for adult and baby leaves (4–5 g/100 g dw), both higher than inflorescences and roots (~2 g/100 g dw). In terms of available carbohydrates, roots presented the highest content (~80 g/100 g dw) compared to the other parts of the plant (30–44 g/100 g dw). According to the European Commission, a fiber-rich food must contain at least 6 g of fiber/100 g or 3 g of fiber/100 kcal [14]. Based on this, all the freeze-dried parts of the plant can be described as sources of fiber, especially adult and baby leaves. In contrast, inflorescences were the richest in total minerals (11 g/100 g dw) and fat (~8 g/100 g) compared to the other samples in study. Furthermore, for a food being considered a source of protein, 12% of its energy value should come from protein. Moreover, to be considered a rich source of protein, this value should be at least 20% (EC, 2006). Based on the protein content obtained for dried adult leaves, they could be also considered an alternative source of protein. 

Our findings differed slightly from those described by Zamorano et al. [15], who reported a lower crude fiber value (12 g/100 g dw) and a higher carbohydrate content (59 g/100 g dw) for *G. tinctoria* leaves. However, ash, fat, and total protein contents were in similar ranges. The differences in carbohydrate and total fiber could be due to differences in several influencing factors, such as degree of leaf maturation, soil, or edaphoclimatic conditions to which the plants were subjected.

### 2.2. Antioxidant Profile

According to the data shown in Table 2, baby leaves present a significantly higher (*p* < 0.05) content of total phenolics (308 mg GAE/g) compared to the other parts of the plant under study. In turn, roots contain the highest amount of total flavonoids (30 mg CE/g). The antioxidant activity of the samples was evaluated using two different methods (FRAP and DPPH^●^ inhibition). Baby leaves showed a considerably higher antioxidant activity (*p* < 0.05), by both methods, compared to the other parts of the plant. In contrast, roots presented the lowest antioxidant activity (*p* < 0.05). No significant differences (*p* > 0.05) were found between the antioxidant activity of adult leaves and inflorescences. Zamorano et al. [15] reported a total of 10.7 g GAE/100 g sample in the case of total phenolics, 111.9 g ascorbic acid equivalents/100 g in the FRAP method, and 83.3 mg TE/100 g in the DPPH^●^ inhibition method, regarding adult leaves of *G. tinctoria*. However, our results cannot be directly compared due to the different extraction methods used. While, in our study, an equitable ethanol/water mixture was used for antioxidant assays, in the study conducted by Zamorano et al. [15] the authors used water with 1% HCl. 

### 2.3. Obtention of Phenolic-Enriched Extracts of G. tinctoria Leaves

#### 2.3.1. Total Phenolic Content

Although baby leaves presented an overall better antioxidant profile, the *G. tinctoria* adult leaves (GuT) were selected to continue the assays, based on their good nutritional profile, good antioxidant activity, and significantly higher abundance in nature, which is of importance considering future applications. 

The sample was extracted with hydroethanolic solvent (GuT_1_) and ultrasonication with water (GuT_2_), being then enriched using Diaion^TM^ HP20LX (Di) and Relite EXA90 (Re) adsorbent resins. The phenolic-enriched fraction of GuT was confirmed based on the total content of phenolic compounds and total flavonoids content, as described in Table 3. The highest extraction yield was achieved with the hydroethanolic extraction (GuT_1,_ 58.0%), and the highest enrichment was obtained with Diaion^TM^ HP20LX (GuT_1_-Di_1,_ 38.6%). Accordingly, the highest flavonoid content was also observed for GuT_1_-Di_1_ (43.1 mg/L). Moreover, total phenolics increased in relation to crude extract (GuT_1_). In regard to GuT_1_-Re_1,_ the enrichment yield was 36.1% and the total phenolics content was 77.8 mg/L with a 2.23-fold enrichment. A significant enhancement in phenolics was also observed for all the other samples under study. However, the lower extraction and enrichment yields obtained in the second series (GuT_2_: 15.3%; GuT_2_-Di_1_: 21.0%; GuT_2_-Re_1_: 22.00%) allowed us to select GuT_1_-Di_1_ as the best sample/process to achieve higher extraction and enrichment yields, and higher richness in phenolics compounds (Table 3). These results are in accordance with those already published by Davoodi et al. [16] in which the phenolic fraction of *Satureja khuzistanica* was purified by Diaion HP20 regarding pre-preparation of phenolic extract for open column chromatography and HPLC to find a new natural scaffold. Phenolic enrichment was also confirmed in another study with a similar protocol using *Punica granatum* peel [17]. The EXA-118 adsorbent resin was also successfully employed in the purification of anthocyanins and hydroxycinnamic acids from a citrus by-product, as well [18]. Additionally, the use of adsorbent resin column chromatography in the purification of food ingredients as a substantial modern source of economic technology was previously proved in food area to obtain high purity end products [19]. 

### 2.4. Bioassays

**Figure 1:**
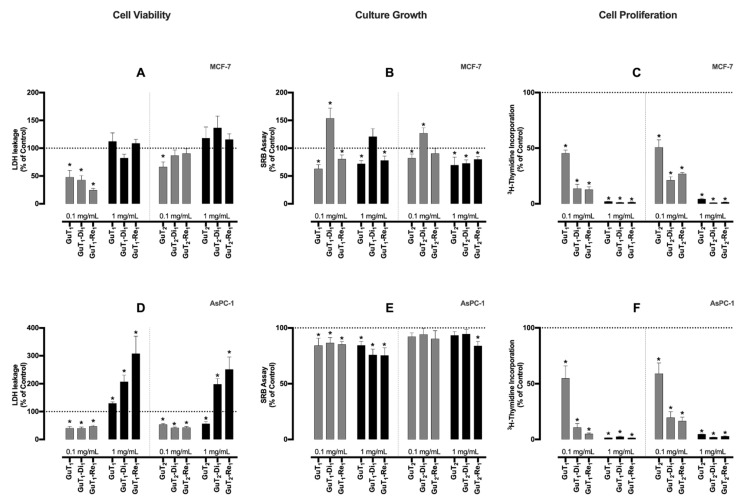
GuT_1_, GuT_1_-Di_1_, GuT_1_-Re_1_, GuT_2_, GuT_2_-Di_1_, GuT_2_-Re_1_ extracts activity on MCF-7 breast (**A**–**C**) and AsPC-1 pancreatic cancer cells (**D**–**F**). Long-term (24 h) effects of the extracts in two different concentrations (0.1 and 1 mg/mL) on cell viability (extracellular LDH activity), culture growth (SRB assay), and proliferation rates (3H-thymidine incorporation) of MCF-7 and AsPc-1 cell lines (*n* = 5–7). Data shows arithmetic means ± S.E.M. * *p* < 0.05 vs control, by Student’s *t*-test.

Gallic acid, ellagic acid, catechin, epicatechin, and quercetin have been described as major phenolics of *G. tinctoria* [8,20], and all of them are widely known for their antioxidant properties. Based on the hypothesis that the phenolic-enriched fraction of *G. tinctoria* would exert antitumoral activities in breast and pancreatic cancer cell lines, their effects on cell viability, culture growth, and proliferation were studied. Considering cell viability (Figure 1A,D), for the lowest concentration, after 24 h, none of extracts was able to exert a cytotoxic activity on both cell lines. Observing the results with the MCF-7 cell line for the highest extract concentration (1 mg/mL), it is possible to note a cytotoxic tendency induced by almost all extracts except GuT_1_-Di_1_, although with no statistical differences compared to control. However, regarding the AsPC-1 pancreatic cancer cell line (Figure 1D), all samples, except GuT_2_ at 1 mg/mL, had a marked significative effect upon cell viability. In particular, GuT_1_-Re_1_ (307% of control) and GuT_2_-Re_1_ (250% of control) exerted a strong cytotoxic activity on this line. Nevertheless, these samples were passed over due to the low enrichment yield and the phenolic content, as described in Section 2.3.1.

Concerning culture growth (by SRB assay), after 24 h, almost all extracts in both concentrations were able to reduce the MCF-7 cell mass (Figure 1B). The reduction in culture growth was more marked by GuT_1_ and GuT_2_ in both concentrations. Data regarding the SRB assay in AsPC-1 cell line have shown that practically all extracts exerted a reduction effect on this cell line mass (Figure 1E), being GuT_1_-Di_1_ (76% of control), GuT_1_-Re_1_ (85% of control), and GuT_2_-Re_1_ (84% of control) extracts more powerful in the highest concentration.

Interestingly, the results of the cell proliferation assays (Figure 1C,F) were the most expressive ones. In both cell lines, the anti-proliferative effect seemed to be concentration-dependent to all extract samples and it was possible to observe a reduction greater than 90% (comparing to control) in all extracts in the highest concentrations. These impressive results show that both crude extracts (GuT_1_ and GuT_2_) and phenolics-enriched fractions could have an important role against cancer, particularly in inhibiting cancer cell proliferation and tumor growth. Most of the observed effects are probably linked to the antioxidant and anti-inflammatory properties of polyphenols [21] especially gallic acid, ellagic acid, catechin, and epicatechin [22] by acting on multiple molecular and cellular targets and may influence the three phases of chemoprevention, namely, primary prevention (inhibition of cancer initiation), secondary prevention (inhibition of cancer promotion), and therapy (inhibition of cancer progression) [23]. 

Considering the results of all the previous steps (product extraction yield, enrichment rate, total phenolics and flavonoids contents, and anti-tumoral activities) both GuT_1_-Di_1_ and GuT_1_-Re_1_ detached as the best fractions. Due to the similarity between them, we selected one (GuT_1_-Di_1_) as a reference sample for the following studies, namely, the preparation of lipid-based micro/nanoparticles protected with natural polymers. 

### 2.5. Physicochemical Properties of Nano/Micro-Particles

**Scheme 1:**
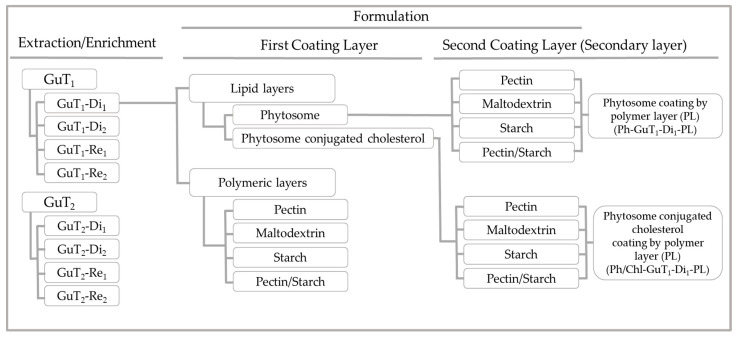
Scheme of sample preparation.

Nano-phytosomes were produced as described in Section 3.7.1. The main objective of this step was to considerably improve the physicochemical properties of the lipid-based nanoparticles leading to the production of nano-phytosomes and phytosomes conjugated with cholesterol shielded by different natural polymers with the best properties to protect the phenolic compounds (Scheme 1). Indeed, the physicochemical properties of a particle such as shape, appearance, flowability, miscibility, and ease of handling play a vital role to achieve the desired performance [24]. For that, different proportion of substrates were tested for coating phytosome layers, and the final particles were compared in terms of production yield, particle size distribution, surface charge, and morphologies. Additionally, the bilayer formation as well as the coating layers variability were monitored regarding the particle’s size distribution of inner layers which play an essential role on functionality. For that, the probable formation of inner layers was discussed and clarified, for first time, in terms of size, surface charge, and particle distribution. Technical analyses like Fourier-Transform Infrared Spectroscopy (FTIR) and molecular docking (for the first time) were also carried out, to confirm the interaction between phenolic compounds and the lipid bilayer.

#### 2.5.1. Formulation Yield

The formulation product yield is determined by subtracting the amount of final powder recovered from the spray dryer collector to the initial amount of feeding solution. This parameter is affected by solution viscosity, volume of feed solution, and feeding temperature, having also into account the type of natural polymer (coating layer) and the drying conditions [25]. In the present work, the inlet temperature was set based on the natural polymer features (Section 3.7.4), ranging between 115 and 130 °C, in order to reduce the moisture in the final product and enhance the formulation product yield by optimizing the inlet and outlet temperatures [26]. The product yields varied between 32.0% and 68.9%, significantly differing according to the type of natural polymer and drying conditions. The following range is in agreement with those of different studies mentioned by Tontul and Topuz in their review paper [25] that explain based on literature data that a considerable variability on product yield is entirely influenced by the type of natural polymer [25]. In this work, the highest product yields were obtained with starch, while the lowest ones were observed for maltodextrin. Our results are in accordance with those of Higuita et al. [27] that reported a high product yield for starch and a low product yield for maltodextrin. It must be taken into consideration that a significant mass loss occurred throughout the drying process due to 100% aspiration by the vacuum system (most of the light and small particles were vacuumed and placed on the outlet filter). Additionally, a certain powder mass was diminished due to adhesion of un-encapsulated particles to the inner surface of the drying cyclone just after nozzle aspiration (drying chamber). The following fact quite justified the low product yield values obtained for some variables [26]. 

In the case of the polymeric layer and Ph-GuT_1_-Di_1_ secondary layer, there were no significant differences between the product yields. The results demonstrate that the secondary layer was following routes like a single polymeric layer, and no considerable changes were observed in terms of product yield compared to polymeric layer. In turn, in the case of Ph/Chl-GuT_1_-Di_1_ secondary layer series, the product yield values were observed in a higher rank compared to other series (53.0–70.2%). More details are presented in Table 4. It could be concluded that the related factor might depend on the impact of cholesterol to improve the stability of lipid particles, and the affinity of cholesterol to a natural polymer originates a more stable particle during the process. Furthermore, incorporating cholesterol in the phytosome layer might result on a strong adhesion between Ph/Chl-GuT_1_-Di_1_ colloidal particles, causing a considerable increase on total product yield. The following is certain: the effect of conjugating cholesterol with the phytosome improved the product yield of the secondary layer, in contrast to the reported by a researcher who explained that the agglomeration and adhesion of particle to the walls of the drying chamber caused a considerable decrease on product yield. Indeed, adhesion of particles on the wall of drying chamber can be influenced by variable factors such as type of carriers, inlet temperature, wall properties, and the feature and percentage of first or binary coating layers [28]. However, our results are in accordance with those of Wang et al. [29] who reported that hydroxypropyl methylcellulose provides a surface coverage of the particles and reduces adhesion during spray drying, resulting on improving the product yield. 

#### 2.5.2. Fourier-Transform Infrared Spectroscopy Analyses (FTIR)

**Figure 2:**
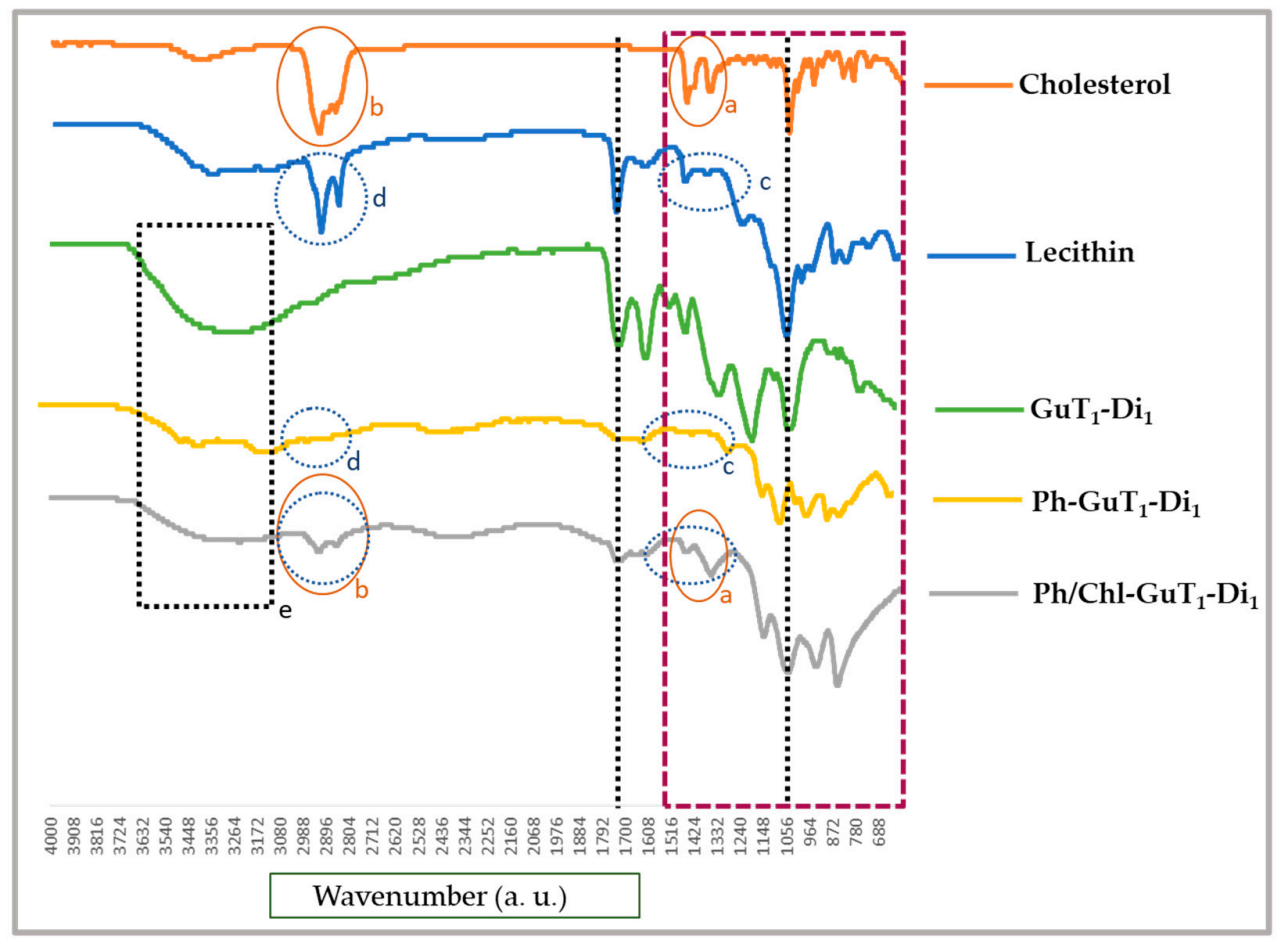
The critical change on functional groups of Ph-GuT_1_-Di_1_, and Ph/Chl-GuT_1_-Di_1_ are clearly marked in FTIR spectra.

The recorded FTIR spectra confirmed the interaction between GuT_1_-Di_1_, lecithin (phosphatidylcholines), and cholesterol in the nano-phytosome structure (Figure 2). The FTIR measurements were performed in the scanning range from 4000 to 400 cm^−1^. The spectra prove the interaction between the lipid carrier and the core material via significant differences between the pure compounds and the complexes. Suppressing of critical functional groups of the substrates which occur in Ph-GuT_1_-Di_1_ and Ph/Chl-GuT_1_-Di_1_ spectra are marked in Figure 2. Furthermore, the significant changes in the fingerprint area 400–1500 cm^−1^ (purple box in Figure 2) of formulated samples clearly shows the existence of phytosome interactions. Moreover, regarding (Figure 2); Peaks (b) marked with orange color at 2930 cm^−1^ appear at the same area of Ph/Chl-GuT_1_-Di_1_. In contrast, there is a lack of existence of this peak in the same area (c) in Ph-GuT_1_-Di_1_ spectra which did not contain cholesterol, confirming the interaction of cholesterol with lecithin in Ph/Chl-GuT_1_-Di_1_. The peaks (d) marked with blue color at 2924 cm^−1^ appear with low intensity on Ph-GuT_1_-Di_1,_ and with high intensity on Ph/Chl-GuT_1_-Di_1,_ confirming the interaction of lecithin in both structures. Furthermore, the suppressed area (e) at Ph-GuT_1_-Di_1,_ and Ph/Chl-GuT_1_-Di_1,_ compared to GuT_1_-Di_1_ confirm the interaction between substrates. The physicochemical interaction between the lipid layer and GuT_1_-Di_1_ as a subsequent phytosome nanoparticle is then confirmed by observing the differences between pure compounds and phytosome complexes [30]. Moreover, suppressing on the sharp endothermic functional groups of curcumin as an active ingredient as well as phospholipid spectra were reported before, which confirm our outcome [31]. 

#### 2.5.3. Particle Size Distribution and Morphology

**Figure 3:**
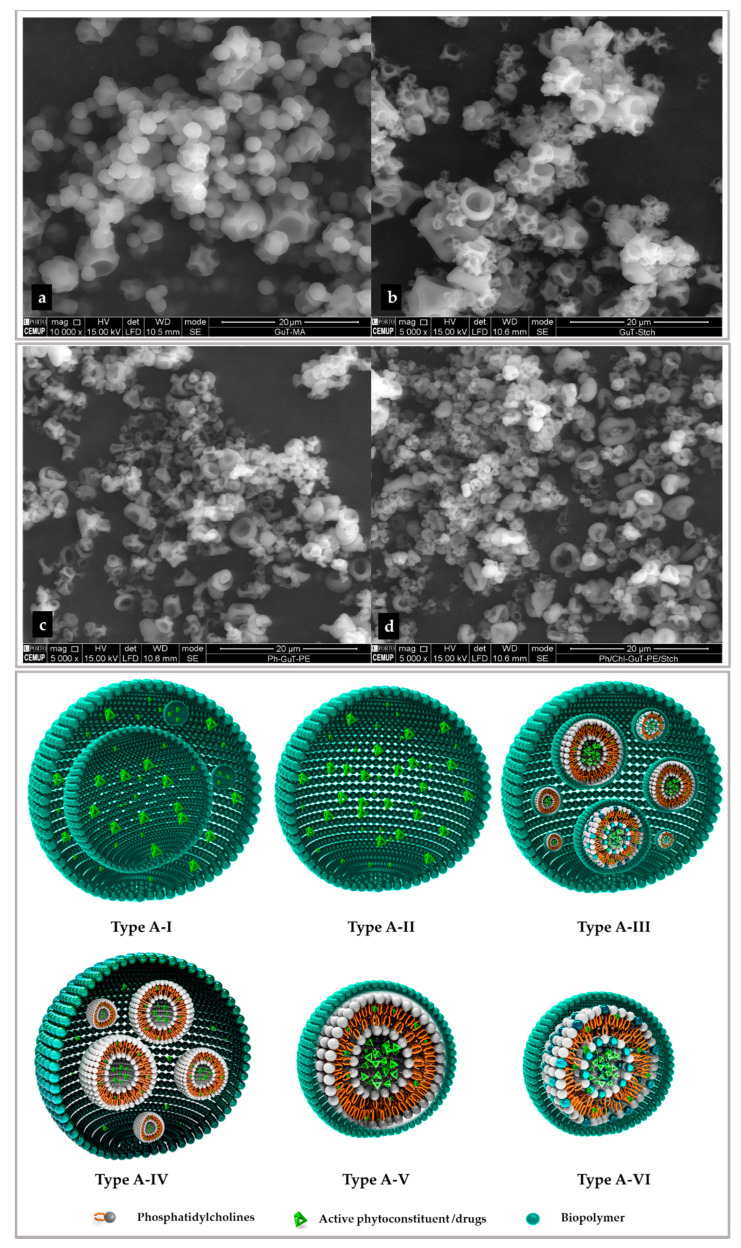
Surface morphology of final products: (**a**) maltodextrin; (**b**) starch; (**c**) pectin; (**d**) pectin/starch.

Particle size was analyzed regarding effective diameter on number and volume. The particle size presented almost at an inharmonious size distribution in all samples. Three types of particles were monitored in the current study: (i) the lipid layer of nano-phytosome (Ph-GuT_1_-Di_1_) and cholesterol conjugated phytosome (Ph/Chl-GuT_1_-Di_1_); (ii) the simple coating of different natural polymers (polymeric layer); and (iii) the polymeric layer coating a both lipid layers individually (secondary layer). For this purpose, the particle sizes of all samples were analyzed regarding the external and internal layers. The particle categories have been listed in Table 4, and more details are schematically explained in Figure 3.

##### 2.5.3.1. Lipid-Based Layers

Particle size distribution was evaluated after 20 min of sonication for Ph-GuT_1_-Di_1_ and Ph/Chl-GuT_1_-Di_1_ due to the preparation method. The Ph-GuT_1_-Di_1_ particle size observed was 33.5 and 120 nm regarding number and volume, respectively, being considered a nano-phytosome. Our result is in accordance with those of Nazari et al. [32] that reported a nano-phytosome with a particle size of 115 nm. The Ph/Chl-GuT_1_-Di_1_ particle size was 140.2 and 354.4 nm, regarding number and volume, respectively, being considered a colloidal particle [33,34]. In comparison, Ph-GuT_1_-Di_1_ showed to produce a smaller particle and the presence of cholesterol made the phytosome bigger. This is in accordance with Rasaie et al. [35], who also reported that a cholesterol conjugated phytosome produced a particle with a size of 393.67 ± 2.89 nm while promoting a significant physical stability on the phytosome layer (particle size stability for more than 20 days).

##### 2.5.3.2. Polymeric Layers

A 7 min sonication was applied before analyzing the particle size to crush the outer layer and detect the size of inner layers (Figure 3). The particle size of the external layer is discussed in Section 2.5.4. The particle size was observed in the range of 327.1–916.5 nm in number and 397.9–1196.5 nm in volume, regarding the inner layer. Pectin, the complex of pectin/starch, starch, and maltodextrin produced the biggest to the smallest particles, respectively, (Table 4). Regarding a considerable protection of pectin as coating layer, finding a strategy to reduce the size is essential [36]. For this purpose, considering the size of starch in simple coating and in the complex of starch with pectin, it could be concluded that starch affected the pectin size and considerably decreased the particle size in consequence of complexation (Table 4). Therefore, based on experimental data, starch acts as a size reducing agent in the complex with pectin. It could be taken into consideration that a carbohydrate like starch link to the surface of polymer layers and prevent the agglomeration due to steric hindrance mechanisms. Therefore, starch could limit the size by affecting the surface of micro/nanoparticles [37], which was proved in the current study, as well.

##### 2.5.3.3. Secondary Layers 

A 5 min sonication was applied before analyzing the particle size to detect the size of inner layers, just like in the previous Section 2.5.3.2 (Figure 3). Polymeric secondary layers coating Ph-GuT_1_-Di_1_ and Ph/Chl-GuT_1_-Di_1_ were quite similar in size distribution. The particles size of these two series were quite bigger compared to polymeric layers. The following result might occur due to aggregation of lipid layers inside one another, creating a bigger particle (Figure 3). The particle sizes of the inner layers regarding the secondary coating layers were observed in the range 226.3–1016.1 nm, in number, and 411.5–1364.6 nm, in volume, for the Ph-GuT_1_-Di_1_ series. In addition, ranges between 212.7 and 1073.0 nm in number, and 392.7–1207.9 nm in volume were observed for Ph/Chl-GuT_1_-Di_1_ series (Table 4). The biggest and the smallest particles were recognized for pectin at 1016 nm in number and 1364 nm in volume, and maltodextrin (212.7 nm in number and 392.7 nm in volume), respectively. It must be reinforced that a significant reduction on particle size of pectin in complex with starch was observed in these, as well. Therefore, it could be a nice prove to consider starch as a size reducing agent [37]. 

#### 2.5.4. Scanning Electron Microscopy (SEM)

SEM images were used to confirm the surface morphology regarding the type of coating layer and particles size, although it could not give any information about the inner layer (Figure 3). Surface morphologies were approximately spherical, crushed sphere, with regular and irregular surface shape for all analyzed particles (Figure 3). The most smooth and spherical surface shape was observed for maltodextrin in polymeric layers and secondary layers. A spherical uniform surface with low concavities was also previously observed for maltodextrin [38]. Starch and pectin in simple matrix as well as in complex have a similar surface shape. Both particles have a regular and spherical surface with some cavity and some small particles seem to be crushed spheres (which might be due to the use of high temperature during preparation). For comparison, pectin has more depth concavities and starch was observed more sphere. Our results are in accordance with those of Cortes et al. [39]. Considering the particle morphology observed by SEM, it was shown that the particle morphology is directly influenced by the type of coating layer and conditions of the drying process [25] and it was not influenced by the type of inner layer. Since the size of the inner layer for most of particles was demonstrated to be in the nanometer range (except for pectin-based particles, Section 2.5.3), it was obviously proved by SEM images that the secondary layer has a bigger size, almost around 1–20 µm (colloidal system [33]) for all samples without exception (Figure 3). This is in agreement with the results published by Kolan et al. [36], regarding maltodextrin and pectin, and Cortes et al. [39], regarding starch and pectin, in terms of particle size and morphology. Furthermore, Figure 3 schematically introduces the probable formation of layers in several particles and demonstrate the multiplate formation of layers which significant influences the particle size. In the case of layer-by-layer encapsulation (coated by natural polymer), if the polymeric layer only encapsulates the active part in normal conditions (polymeric layer) the particle inner layer might get shaped similar to image structure without any internal layer: Type A-I or Type A-II with some internal layers. In the case of phytosome coated with a polymer, the particle inner layers appear as Type A-III, A-IV, A-V, and A-VI or a combination of all. In structure Type A-II, A-III, and A-IV some agglomerations of small particles with big dimensions cause these structures. It must be noticed that a combination of all these structures occurs typically in the total sample (Type A-IV). The type of layer could be approximately estimated based on a gap between volume and number for each particle. When the difference between a volume and number is low the structure Type A-II was formed. When the difference between a volume and number is high, some structures (Type A-III, and A-IV) occur. Moreover, when the particles size in both number and volume are quite near to the size of nano phytosomes, it shows that the images Type A-V and A-VI are present. In addition, by optimizing preparation conditions, some of these categories might decrease, although it is not possible to eliminate them. Hence, coated particles are formed into different main structures that directly influence the size, sequence, and shape of particle layers. 

#### 2.5.5. Zeta Potential (Surface Charge)

The particles surface charge/zeta potential (ZP) was measured for all samples regarding the lipid layer, inner layers, polymeric layer, and secondary layer. Commonly, surface charge can directly affect a cellular permeate tendency due to cellular membranes surface charge. A surface charge greater/lower than ±30 mV is desirable in terms of cationic/anionic stability at aqueous media since it induces particle repulsion and diminishes agglomeration. Furthermore, if surface charge is within ±10 mV the particle is considered neutral. The range between ±10 mV and ±30 mV is adjusted to the nearest charge tendencies. It must be noted that the cationic particle provides more toxicity associated with cell wall membrane [40,41]. As expected, the phytosome particles were observed at the negative charge of (−67.50 ± 1.43 mV) and (−62.57 ± 2.95 mV) associated to Ph-GuT_1_-Di_1,_ Ph and Chl-GuT_1_-Di_1,_ respectively. The anionic charge is related to the layer of phosphatidylcholine (lecithin) and cholesterol. The negative charge of lipid phytosome layer has been also reported previously with slight differences [42,43]. Ph-GuT_1_-Di_1,_ Ph and Chl-GuT_1_-Di_1_ showed a zeta potential greater than 60 in anionic mode, suggesting a high stability of the nanoparticle in solution media, while the results published in literature were lower and consequently represent a lower stability [40,41]. 

The surface charge of polymeric and secondary layer was observed in negative charge even in inner and external layer with small differences in surface charge in the ranges from −21.26 ± 7.19 to −55.01 ± 3.24, associated to inner layer, and from −14.99 ± 2.79 to −53.47 ± 1.94, associated to external layer, for all samples, in general. The negative ZP of pectin, maltodextrin, and starch was confirmed previously [44,45]. To a large extent, the surface charge of inner layer presented a higher value, which proved that the inner layers are considered desirable in terms of stability due to the negative electric charge that induce particle repulsion and reduce agglomeration in solution media. Typically, a higher surface charge promotes a higher colloidal stability and lower particle agglomeration [46]. Although the stability of inner layer was considered more desirable, the external layer was also in an acceptable range [40]. As a result, particles in all categories were observed in acceptable negative charge mood except pectin in terms of inner layer, both on polymeric and coating layers which showed the lowest values (Table 4). Although the following value are not adjusted on best range, there are suited to negative surface charge tendencies [40]. However, a size reducing tendency of starch in the mixture of pectin/starch was demonstrated in Section 2.5.3.1, Section 2.5.3.2 and Section 2.5.3.3, showing a relative feature on ZP, as well. The stability of pectin was improved when combined with starch regarding the ZP value, in all related particles (Table 4). Concluding, the complex of pectin with starch can not only be considered a size reducing agent, but also a particle stabilizer agent leading to greater mechanical resistance in aqueous media. A considerable enhance on surface charge of pectin/starch complex was shown in all experimental samples. It must be taken into account that the physiochemical properties of the inner layer such as particle size, zeta potential, and, generally, its availability has not been focus of attention in any related research until now. Therefore, in this study, for the first time, the inner layer formation is clarified and discussed, regarding physiochemical properties as particle size and zeta potential.

### 2.6. In Silico Investigation Based on Molecular Arrangement

**Figure 4:**
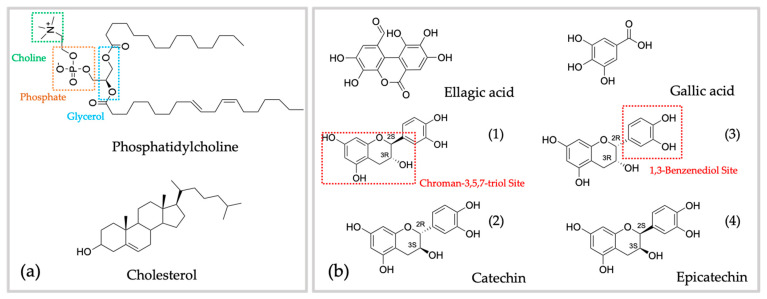
Chemical structure of lipid layer constituents (**a**) and phenolic compounds previously identified in *G. tinctoria* (**b**). *Regarding catechin* (1); (3*R*)-2(*S*)-(3,4-dihydroxyphenyl)chromane-3,5,7-triol, (2); (3*S*)-2(*R*)-(3,4-dihydroxyphenyl)chromane-3,5,7-triol, (3); (3*R*)-2(*R*)-(3,4-dihydroxyphenyl)chromane-3,5,7-triol, (4); (3S)-2(S)-(3,4-dihydroxyphenyl)chromane-3,5,7-triol.

**Figure 5:**
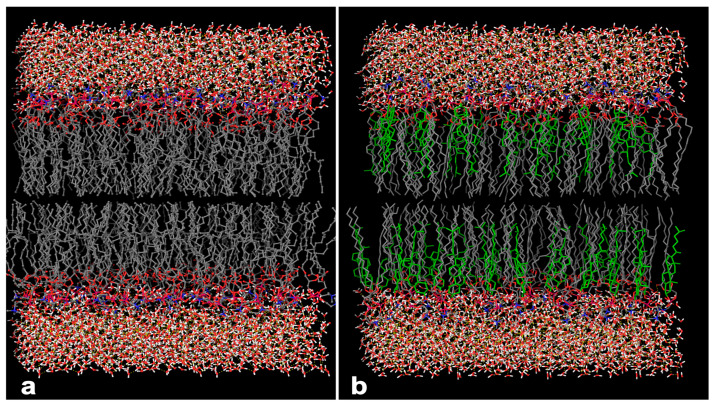
Simulated 3D model of lipid bilayer. (**a**) Nano-phytosome prepared using lecithin; the polar site of phosphatidylcholine is represented with red color and the aggregation of white and red molecules at the top and bottom of the bilayer grid exhibit the water molecules the surround the lipid bilayer. (**b**) Phytosome prepared using lecithin conjugated with cholesterol; the green structure shows the arrangement of cholesterol within phosphatidylcholine.

Gallic acid, ellagic acid, catechin, epicatechin, and quercetin (Figure 4) have been described as major phenolics of *G. tinctoria* [8,20], and all of them are widely known for their antioxidant properties which were discussed on (Section 2.4), as well. Considering the high content of phenolic compounds identified in our samples (Table 3), in silico investigation and molecular arrangement of phospholipids was evaluated by molecular docking to confirm the incorporation of phenolic compounds in the simulated lipid bilayer. The represented 3D model of lipid bilayers (Figure 5) was constructed using the MemGen webserver. The selected phenolic compounds docked with both constructed bilayer and cholesterol conjugated phytosome according. The low docking score was attributed to have more hydrogen binding and stronger interactions [47]. Table 5 presents the docking Score and grid energy in detail. 

**Figure 6:**
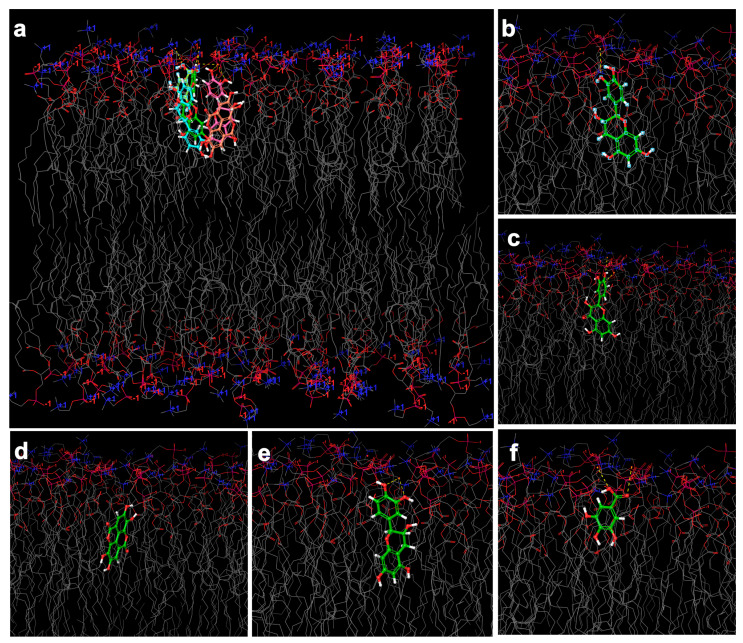
Simulated 3D model of the nano-phytosome bilayer covered by thousands of water molecules (the red structure in the top and bottom). In real media, the bilayer appears as spherical, elliptical, or as a structure between both. The grid section of nano-phytosome bilayer is shown. The molecular docking images present the phenolic compound placed within the nano-phytosome bilayer structure with a number of hydrogen bindings; (**a**) overlaid of all compounds interaction with the bilayer, (**b**) catechin, and (**c**) epicatechin; both interacted through the 1,3-benzenediol site with the polar site of lipids with lower docking score and stronger hydrogen binding. (**d**) Ellagic acid, (**e**) quercetin, (**f**) gallic acid.

The docking score result revealed the hydrogen binding between the polar site of phenolic compounds and the choline site of phosphatidylcholine. For example, a significant number of hydrogen bonds between quercetin hydroxyl groups with polar bilayer parts revealed a lower docking score and stronger interaction. This phenomenon may be due to two different factors: size and polarity of the docked molecule which was joined and created the same feature with higher intensity. Considering the structural similarity between catechin, epicatechin, and quercetin, the interaction mode and binding energy are estimated to be similar. However, the rigid structure of quercetin regarding the lack of chiral center may cause a more potent binding energy. Nonetheless, the predominate glucosides formation of quercetin at natural sources may change the docking score due to their conjugated molecule and functional group (Figure 6, Table 5). 

**Figure 7:**
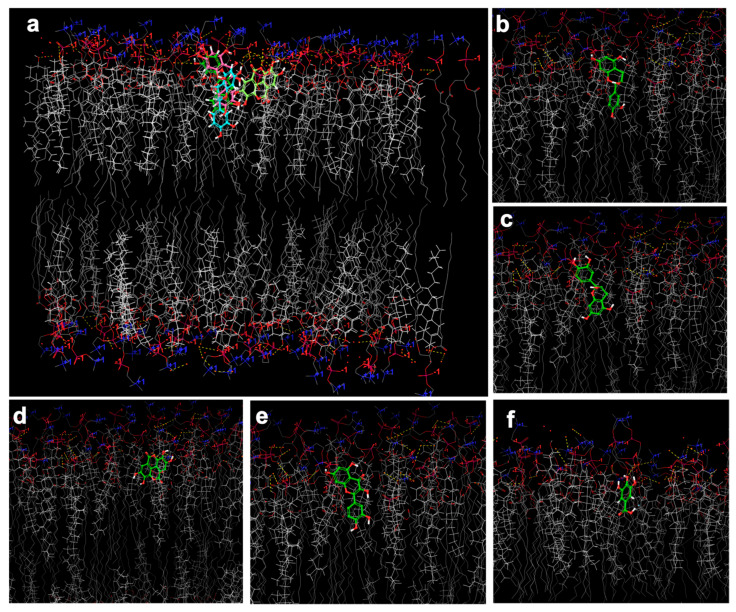
Simulated 3D model of cholesterol-conjugated phytosome docked by phenolic compounds. The phenolic structure is placed within the cholesterol bilayer with a number of hydrogen bindings; (**a**) overlaid of all compounds interaction with bilayer, (**b**) catechin; the hydrogen binding occurred between the chroman−3,5,7-triol site and the polar site of phosphatidylcholine (phosphate and choline sites) and the hydroxyl group of cholesterol, and the 1,3-benzenediol site of catechin was adjusted approximately near to the tail, (**c**) epicatechin; the hydrogen binding occurred between the 1,3-benzenediol site of epicatechin and the polar site of phosphatidylcholine and cholesterol. In addition, the chroman−3,5,7-triol site was adjusted near the tail. (**d**) Ellagic acid, (**e**) quercetin, (**f**) gallic acid.

Regarding the cholesterol-conjugated phytosome, cholesterol was adjusted approximately at the same level of phosphatidylcholine (Figure 4). The hydrogen binding might occur between the phosphate site of phosphatidylcholine and the 3-hydroxy substituent of cholesterol, creating the stability on lipid bilayer that was mentioned on Section 2.5.1, regarding product yields. Additionally, a considerable increase on the size of Ph/Chl-GuT_1_-Di_1_ compared to Ph-GuT_1_-Di_1_ (Section 2.5.3.1, Table 4) was clarified regarding the specific molecular arrangement of cholesterol on lipid bilayer shown in Figure 4 and Figure 7. 

Docking score was recorded in lower value attributed to higher binding energy in all samples belong to phytosome conjugated cholesterol compared to phytosome structure in the same conditions except gallic acid. However, ellagic acid adjusted entirely to the choline site of phosphatidylcholine and to the 3-hydroxy substituent of cholesterol by multiple hydrogen binding (Figure 7). 

In contrast, the molecular arrangements of catechin and epicatechin were quite different at the grid of the cholesterol-conjugated phytosome compared to nano-phytosome bilayer. Catechin and epicatechin were entirely adjusted in the opposite molecular arrangement (Figure 5 and Figure 6). It was shown that the hydrogen binding between the polar site of phosphatidylcholine and cholesterol with 1,3-benzenediol of epicatechin was strengthening this architecture compared to catechin (Figure 4 and Figure 7). In the case of quercetin, it could be concluded that the hydrogen binding between chroman−3,5,7-triol with the polar site of phosphatidylcholine and the hydroxyl group of cholesterol create the strongest interaction compared to all the sites of action under study. Our results confirm the idea of Ghanbarzadeh et al. [48], who demonstrated the occurrence of hydrogen binding between the hydroxyl group of the catechin and the polar site of phosphatidylcholine (phosphate group) in catechin loaded nano-phytosome.

It could be concluded that the polar group is adjusted in the upper head side of phospholipid (polar site) or any type of lipid layer, and the nonpolar structure is placed near the tail. Therefore, a variety of active compounds, in this case phenolic structures, provide a diversity on molecular arrangement of lipid bilayers. Based on the provided information, the polarity of the active compound is a major key factor in the molecular arrangement of lipid bilayer, and the molecule size placed in the second rank. Moreover, taken into consideration the results obtained from molecular docking, the structural arrangement of the nano-phytosome layer was confirmed and it was in accordance with previous research in the area of liposome and phytosome that reported that active structures are placed in core or lipid layer based on the polarity of the active phytoconstituents/drugs, in the case of liposome [49], and adjusted just in polar site in the case of phytosome [48] However, the existence of hydrogen bonding in the phytosome layer was confirmed in this study which is in accordance with Ghanbarzadeh et al. [48], who reported that the chemical bonding also occurs between the active substrate and phospholipids in the phytosome structure. Additionally, it might be more accurate to confirm the target delivery of lipid layer by molecular docking. Concluding, the features could be easily adjusted to all type of lipid layers for future research. 

## 3. Materials and Methods

### 3.1. Chemicals and Reagents

Ferrous sulfate heptahydrate, Folin-Ciocalteu’s reagent, 2,2-diphenyl-1-picrylhydrazyl radical (DPPH), sodium nitrite, ferric chloride, aluminum chloride, 2,4,6-tripyridyl-s-triazine (TPTZ), sodium acetate, trolox, gallic acid, chlorogenic acid, (±) catechin hydrate, and an enzyme kit (α-amylase, protease, and amyloglucosidase) were all supplied from Sigma Chemical Co. (St. Louis, MO, USA). Anhydrous sodium carbonate was from VWR International (Leuven, Belgium).

Sodium hydroxide, Kjeldahl catalyst tablets (Na_2_S_2_O_8_/CuSO_4_), anhydrous sodium sulfate, and concentrated sulfuric acid 98% were from Merck (Darmstadt, Germany). Petroleum ether and ethanol were from Honeywell (Saint-Germain-en-Laye, France). Boric acid was from Panreac (Barcelona, Spain). Acetone, sodium hydroxide, and hydrochloric acid were from Carlo Erba (Val de Reuil, France).

^3^H-thymidine ([methyl-^3^H]-thymidine; specific activity 79 Ci/mmol) (GE Healthcare GmbH, Freiburg, Germany); antibiotic/ antimycotic solution (100 U mL^−1^ penicilin, 100 µg mL^−1^ streptomycin, and 0.25 µg mL^−1^ amphotericin B, HEPES (N-2-hydroxyethylpiperazine-N′-2-ethanesulphonic acid), NADH (nicotinamide adenine dinucleotide), sulphorhodamine B, trichloroacetic acid (TCA), Tris, triton X-100, and trypsin-EDTA solution (Sigma, St. Louis, MO, USA); fetal bovine serum (Invitrogen Corporation, Waltham, MA, USA).

The ion exchange resin Relite EXA90 (Re), and the spherical porous polystyrene resin Diaion^TM^ HP20LX (Di) were kindly gifted from Resindion group of Mitsubishi Chemical, Binasco, Italy. Ethanol with purity of ≥99.8%, 78%, and 96% and dichloromethane were supplied from Honeywell, Germany. Absolute ethanol was purchased from (Loughborough, UK). Sodium phosphate dibasic heptahydrate (Na_2_HPO_4_·7H_2_O) and sodium phosphate monobasic monohydrate (NaH_2_PO_4_·H_2_O) were purchase from PanReac, Barcelona, Spain. Maltodextrin was purchased from Fargon, Barcelona, Spain. Apple pectin was supplied from LABChem, Portugal. Potato Starch (water soluble) was supplied from PanReac-Applichem, Darmstadt, Germany. Cholesterol was supplied from SIGMA life science, USA. Soybean lecithin was purchased from Alfa Aesar, Thermofisher, Kandel, Germany. The ultra-pure water was obtained from PRO 60 CN and Seradest (water purification system).

### 3.2. Plant Material

Roots, inflorescences, baby leaves, and adult leaves of *G. tinctoria* were collected in Sete Cidades, São Miguel Island, Azores, Portugal. The baby leaves were collected when they were 3 to 6 cm long, while adult leaves presented about 50 cm of diameter. Collected roots had approximately 20 cm length, and inflorescences up to 40 cm in length. 

The samples were frozen at −80 °C and freeze-dried (TELSTAR, Cryodos freeze dryer, Barcelona, Spain). The lyophilized samples were powdered and stored in vials. The ground freeze-dried adult leaves had a texture similar to wheat flour, while the baby leaves presented a fluorescent green texture and smell similar to tea leaf. In turn, ground freeze-dried inflorescence had a fibrous non-uniform texture while the roots presented a fibrous uniform texture.

### 3.3. Nutritional Composition

#### 3.3.1. Moisture Content

The moisture content was determined by KERN infrared lamp, DBS60-3. Briefly, 2–3 g of sample were dried at 105 °C in triplicate. The results are expressed in g of moisture per 100 g of sample.

#### 3.3.2. Ash Content

The ash content was determined by incineration (0.6 g of sample) at a muffle furnace heated gradually from 110 °C to 500–550 °C (Thermolyne 48000, Electrothermal Engineering Ltd., Essex, UK), until white ashes obtention (AOAC 920.153) [50]. The ash content was determined by differences in mass content before and after incineration. Triplicate analyses were conducted, and the results are expressed in g of ash per 100 g of sample.

#### 3.3.3. Protein Content

The protein content was determined with quantification of total nitrogen using the Kjeldahl method (AOAC 928.08) [50]. Briefly, a small amount of sample (0.5–1.0 g) was weighed on nitrogen-free paper and placed in a Kjeldahl tube, along with 20 mL of concentrated sulfuric acid (H_2_SO_4_, 98%) and two catalyst tablets (Kjeldahl tablets). Acid digestion was performed in an automatic digester (K-438, Büchi^®^, Büchi Labortechnik AG, Flawil, Switzerland). An amount of 90 mL of sodium hydroxide (NaOH, 32%) was added to the digested to release ammonia, which was distilled and collected in 60 mL of boric acid (H_3_BO_3_ 4%) at pH 4.65. The Process were operated according to Büchi Labortechnik AG, 2007. The solution was then titrated with H_2_SO_4_ (0.2 M) using methyl red as indicator. Total nitrogen content was multiplied by the conversion factor 6.25 [51,52] to estimate total protein content. The analyses were done in duplicate, and the results expressed in g protein per 100 g of sample.

#### 3.3.4. Fat Content

The Soxhlet method was used for determination of total fat content [50]. Briefly, 5 g of sample was mixed with anhydrous sodium sulfate (Na_2_SO_4_) and placed into cellulose cartridges. Extraction was carried out with petroleum ether during 8 h and, afterwards, the solvent was evaporated and collected. Then, the residue was dried at 100 °C until a constant mass value was obtained. The analyses were performed in triplicate and the results are presented in g of fat per 100 g of sample.

#### 3.3.5. Total, Soluble, and Insoluble Fiber Content

Total fiber content was determined using an enzymatic-gravimetric technique (AOAC 991.43) [50]. Briefly, 0.5 g of sample were mixed with 100 µL of α-amylase and 50 mL of phosphate buffer for 20 min at 95 °C. After, 10 mL of NaOH (0.275 M) were added, adjusting the pH to 7.5 ± 0.2, and 100 μL of protease were added, followed by incubation at 60 °C for 35 min, under stirring conditions. Then, an amount of 10 mL HCl (0.325 M) was added, adjusting the pH to 4–4.6. After adding 100 μL of aminoglucosidase, a new incubation at 60 °C for 35 min was performed. At room temperature, 200 mL of ethanol 96% were added to precipitate soluble fiber. The residue was filtered, washed and dried at 105 °C overnight. Insoluble fiber was determined by a similar procedure but without adding ethanol 96%. Soluble fiber was determined by difference. The results are expressed in g per 100 g of sample.

#### 3.3.6. Available Carbohydrates 

The content of available carbohydrates was determined according to the Equation (1) [52]. The results were presented in g per 100 g of sample.

(1) Available carbohydrates%=100%−(Protein%+Fat %+Ash %+Total Fiber %).

### 3.4. Antioxidant Profile 

Spectrophotometric methods were used for determination of total phenolics and total flavonoids contents and antioxidant activity (by DPPH^●^ inhibition and FRAP assays) [53]. Extractions were performed in triplicate using water: ethanol (1:1) as solvent. The extraction was carried out on a hot plate at 40 °C with magnetic stirring for 60 min. The obtained extracts were filtered and frozen at −20 °C until analysis.

#### 3.4.1. Total Phenolics Content

Total phenolics content was determined as described by Costa et al. [53]. Briefly, 150 µL of Folin–Ciocalteu reagent (1:10) and 120 µL of 7.5% *m*/*v* sodium carbonate were added to 30 µL of extract, followed by incubation at 45 °C for 15 min. Absorbance was monitored at 765 nm (BioTek, Synergy HT, PMT 49984, US). The calibration curve was calculated based on gallic acid standard, with linearity ranging between 5 and 100 mg/L, (R^2^ = 0.9998). Analyses were performed in triplicate and the results expressed in mg of gallic acid equivalents (GAE) per g of sample.

#### 3.4.2. Total Flavonoids Content

Total flavonoids content was determined as described elsewhere [53]. One mL of extract was mixed with 4 mL of distilled water and 300 μL of 25% sodium nitrite. After 5 min at room temperature, 300 μL of 10% aluminum chloride were added, and after another min, 2 mL of 1 M sodium hydroxide solution and 2.5 mL of distilled water were also added. Absorbance was measured at 510 nm (BioTek, Synergy HT, PMT 49984, US). Catechin was used as standard for calibration purposes (2.5–400 mg/L; R^2^ = 0.9996). Analyses were performed in triplicate and the results reported in mg catechin equivalents (CE) per g of sample.

#### 3.4.3. Ferric Reducing Antioxidant Power

The ferric reducing antioxidant power (FRAP) was determined According to Costa et al. [53]. The FRAP reagent (0.3 M acetate buffer; 10 mM TPTZ and 20 mM ferric chloride) was added to an extract aliquot and the mixture was incubated for 30 min at 37 °C, stored in dark place. Absorbance was measured at 595 nm (BioTek, Synergy HT, PMT 49984, US). The results were calculated using a calibration curve with ferrous sulfate (25–500 mg/L; R^2^ = 0.9998). Analyses were performed in triplicate and the results expressed in mmol of ferrous sulfate equivalents (FSE) per g of sample.

#### 3.4.4. DPPH^●^ Scavenging Ability

DPPH^●^ scavenging ability was determined according to method described by Costa et al. [53]. An amount of 270 μL of a DPPH^●^ solution (6 × 10^−5^ M) was added to 30 μL of extract and the reaction was monitored by 40 min at 525 nm (BioTek, Synergy HT, PMT 49984, US). Trolox was used to prepare the calibration curve (5–175 mg/L; R^2^ = 0.9997). Analyzes were performed in triplicate and the results expressed in mg of trolox equivalents (TE) per g of sample.

### 3.5. Obtention of Phenolic-Enriched Extracts of G. tinctoria Leaves

Phenolics extraction was carried out using two different methodologies for comparison: a hydroethanolic maceration (GuT_1_) and an ultrasonication extraction (GuT_2_). 

The hydroethanolic extraction was carried out as follows. The sample (100 g) was macerated, in triplicate, with different and subsequent ethanol: water mixtures (100:0, 50:50, 50:70, and 0:100; 2 L each). Maceration was performed for 24 h for each solvent. The four extracts were combined and ethanol was removed at 40 °C using a rotating vacuum evaporator. The concentrated aqueous extract was stored at 4 °C prior to the next step. 

Ultrasonic extraction was carried out based on the procedure described by Puga et al. [54] but with modifications. The extraction was operated, in triplicate, using 100 g of sample macerated for 2 h with 2 L of deionized water using an ultrasonic probe (BANDELIN electronic, UW 50, Germany) for 30 min, at 25 °C. The solution was then filtered and concentrated at 40 °C using a rotating vacuum evaporator. The concentrated aqueous extract was stored at 4 °C prior to the next step.

Phenolics enrichment was performed according to the methodology described and scaled up at Medicinal Plants and Drugs Research Institute-Shahid Beheshti University [16] using Diaion^TM^ HP20LX and Relite EXA90 adsorbent resin columns (50 cm × 6 cm), separately. Both columns were activated with ethanol ≥99.8% for 12 h. The solvent was then removed and each column was washed five times with distilled water. A concentrated aqueous extract (GuT, 6 g) was diluted in distilled water (500 mL) and loaded into the resin column at a 5 mL/min flow rate and kept for 30 min until all phenolic structures were adsorbed in the resin porous. The column was washed with 5 L of distilled water to remove undesirable compounds such as sugars and chlorophyll; this solution was discarded. Afterwards, the column was eluted with ethanol ≥ 99.8% (2 L) for phenolics desorption. Ethanol was removed using a rotating vacuum evaporator and the enriched phenolic fraction of *G. tinctoria* leaves was freeze-dried (TELSTAR, Cryodos freeze dryer, Spain) [17,55]. 

#### Total Phenolic Content

Total phenolic content, in this case, was evaluate regarding GuT_1,2_ and enriched GuT_1,2_ (Table 3). The procedure was done according to Section 3.4.1. The extracts were diluted (1:1000) beforehand due to high phenolic content.

### 3.6. Bioassays 

#### 3.6.1. Cells and Cell Cultures 

Two types of cell lines were used to perform bioassays. The MCF-7 breast cell line (an estrogen receptor (ER)-positive human breast epithelial adenocarcinoma cell line; ATCC HTB-22; passage numbers 30–38) and the AsPC-1 pancreatic cell line (a human pancreatic cell line with high metastatic rate; ATCC CRL-1682; passage numbers 36–42). MCF-7 and AsPC-1 cells were from ATCC (Manassas, VA, USA).

Cells were maintained in a humidified atmosphere of 5% CO_2_-95% air and were grown in RPMI 1640 (catalogue #R6504, Sigma) supplemented with 2 mM L-glutamine, 1 mM sodium pyruvate, 10% heat-inactivated FBS and 1% antibiotic/antimycotic (MCF-7 and AsPC-1 cells). The culture medium was renewed every 2–3 days, and the culture was split every 8 days. For the determination of viability, proliferation and growth, cells were seeded on 24-well culture dishes (2 cm; Ø 16 mm; TPP^®^, Trasadingen, Switzerland) and used at 80–90% confluence. 

To test the effects of the extracts on cell viability, culture growth and proliferation, cells were exposed to these compounds for 24 h in serum-free culture medium. The concentration of each extract to be tested was selected based on literature and previous works [56,57]. Tested extracts were dissolved in 30% ethanol (ratio *v*/*v*: 70% water: 30% ethanol). The final concentration of solvent in culture media was 1% (*v*/*v*). The final extract concentrations used were 0.1 and 1 mg/mL (*w*/*v*). The control was run in the presence of the solvent.

#### 3.6.2. Cell Viability

Cells were exposed to extracts for 24 h (0.1 and 1 mg/mL). After this period, cellular leakage of lactate dehydrogenase (LDH) to the extracellular culture medium was determined, as previously described [58]. LDH activity was expressed as the percentage of extracellular activity in relation to total cellular LDH activity. 

#### 3.6.3. Cell Growth

Cells were exposed to extracts for 24 h (0.1 and 1 mg/mL). At the end of treatment, culture growth was determined by the sulforhodamine B (SRB) assay, which reports on intracellular protein content, as previously described [58]. 

#### 3.6.4. Evaluation of Cell Proliferation 

Cells were exposed to extracts (0.1 and 1 mg/mL) or vehicle for 24 h, and cell proliferation rates were determined by a 3H-thymidine incorporation assay, as described [56]. DNA synthesis rate was evaluated by quantification of incorporation of 3H-thymidine (mCi/mg total protein) [56]. Intracellular radioactivity was measured by liquid scintillation counting (LKB Wallac 1209 Rackbeta, Turku, Finland). The results were normalized for total protein content (Bradford method) [59]. 

### 3.7. Particles Production

#### 3.7.1. Preparation of Nano-Phytosome and Cholesterol-Conjugated Phytosome

The phytosome preparation followed a thin film hydration method with small modifications for nano-phytosome and cholesterol-conjugated phytosome, both loaded by the enriched phenolic extract [60,61]. 

In case of the nano-phytosome, lecithin (containing 90% soybean; L-α-Phosphatidylcholine) with an optimum molar ratio of (1:1) relative to GuT_1_Di_1_was used as a lipid layer. A total of 50 mg of lecithin was primarily dissolved on 2 mL of dichloromethane and vortexed (Shaker & Mixers Reax top, Heidolph, Germany) for 5 min, at room temperature, until a transparent yellow solution was obtained. A total of 50 mg of GuT_1_-Di_1_ was dissolved on 20 mL of ethanol at 55 °C. The solution of lecithin-dichloromethane was added to GuT_1_-Di_1_ in ethanol and refluxed under stirred conditions, for 2 h, at 55 °C, until a transparent solution was obtained. The solution was cooled down to 37 °C and a thin layer was formed using a rotary vacuum evaporator (37 °C; 60–200 rpm; 55 mbar of vacuum pressure). Then, N_2_ was flushed on the thin layer for 1 min and the flask was sealed and kept overnight in a desiccator [42]. The thin layer was then hydrated with phosphate buffer (pH 5.5), at a vacuum pressure of 200 mbar, 200 rpm, and 40 °C, for 10 min. The nano-phytosome (Ph-GuT_1_-Di_1_) was subjected to ultra-sonication for 25 min, at 60% amplitude, in pulsation mood (5:1 s) [60,62]. 

In turn, the cholesterol-conjugated phytosome was prepared using lecithin and cholesterol loaded with GuT_1_-Di_1_ with an optimum molar ratio of 1:0.5:1, and fabricated using the above described procedure for nano-phytosome with some differences. Lecithin was added to GuT_1_-Di_1_ and stirred for 10 min. Then, the cholesterol solution was prepared just like lecithin solution on dichloromethane and sprayed into the mixture of lecithin/GuT_1_-Di_1,_ followed by reflux under stirred conditions for 2 h, at 55 °C. At the end, the solvent was evaporated, and a thin film was obtained using the same above mentioned condition. The cholesterol-conjugated phytosome (Ph/Chl-GuT_1_-Di_1_) was hydrated and ultra-sonicated using the same referred conditions, as well.

#### 3.7.2. Polymeric Layer

GuT_1_-Di_1_ was employed to load a 10% (*w*/*v*) natural polymeric layer based on the solution viscosity and efficiency for feeding the spray dryer (mini spray-dryer B-290 from BÜCHI (Flawil, Switzerland). Maltodextrin, pectin, and starch, as simple matrixes, and a pectin/starch (1:1) complex were used for biopolymer coating. Four different polymeric solutions with 1% (*w*/*v*) of GuT_1_-Di_1_ in ultrapure water were prepared and stirred for 2 h at 55 °C. The solutions were then injected into the spray dryer under the specific conditions described in Section 3.7.4 [63]. 

#### 3.7.3. Secondary Layer

The secondary layer (binary coating or biopolymer-coated) was prepared with 10% (*w*/*v*) of natural polymer, for Ph-GuT_1_-Di_1_, and 15% (*w*/*v*) of natural polymer, for Ph/Chl-GuT_1_-Di_1_. For this purpose, the natural polymer or the complex (described in Section 3.7.2) was added to Ph-GuT_1_-Di_1_ and Ph/Chl-GuT_1_-Di_1_ immediately after their preparation according to the described order:

- GuT_1_-Di_1_, lecithin, biopolymer (individual) (1:1:0.5:10);
- GuT_1_-Di_1_, lecithin, biopolymer (complex) (1:1:0.5:5:5);
- GuT_1_-Di_1,_ lecithin, cholesterol, biopolymer (individual) (1:1:0.5:15);
- GuT_1_-Di_1_, lecithin, cholesterol, biopolymer (complex) (1:1:0.5:7.5:7.5).

For that, natural polymers were dissolved on ultrapure water and stirred for 2 h at 55 °C. After the solutions were mixed, Ph-GuT_1_-Di_1_/natural polymer and Ph/Chl-GuT_1_-Di_1_/natural polymer were subjected to ultra-sonication for 5 min, at pulsation mode, and stirred for 20 min. The solutions were then injected into the spray dryer to obtain particles in the form of powder [63]. 

#### 3.7.4. Spray-Drying Conditions

The polymeric layer and the secondary layer were dried using a mini spray-dryer B-290 from BÜCHI (Flawil, Switzerland) with a standard 0.5 mm nozzle. The spray drying conditions were optimized regarding the feature of polymers and solutions viscosity. All solutions were prepared in ultrapure water and injected at a flow rate of 10 mL/min, aspiration 100% (36 m^3^/h), air pressure of 5.5–6 bar, and the nozzle cleaner was set to 3. 

The inlet temperature was differently selected according to the type of the natural polymer used. For starch and pectin/starch, the inlet temperature was 130 °C and the outlet temperature was 63 ± 3 °C. In the case of pectin, the inlet temperature was 120 °C and the outlet temperature 58 ± 2 °C. The inlet temperature for maltodextrin was 115 °C while the outlet temperature was 55 ± 2 °C. Throughout the drying process, the emulsion was continuously agitated with a magnetic stirrer, at room temperature, to avoid aggregation of its solid content. Finally, the powders were recovered from the collector and stored in falcon tubes, sealed, and stored at 4 °C. The product yield (%) was calculated by the amount of the powder recovered from the collector and the total mass content of the initial feed solution (Equation (2)) [64,65].

(2) $$Yield=\;\frac{\mathrm{Total}\;\mathrm{weight}\;\mathrm{of}\;\mathrm{final}\;\mathrm{powder}}{\mathrm{Total}\;\mathrm{weight}\;\mathrm{of}\;\mathrm{initial}\;\mathrm{feed}}\;\times \;100.$$

### 3.8. Physicochemical Properties of Nano/Micro-Particles

#### 3.8.1. Fourier-Transform Infrared Spectroscopy Analyses (FTIR)

The formation of phytosome and cholesterol-conjugated phytosome, regarding the existence of interactions between lecithin and cholesterol with GuT_1_-Di_1,_ was evaluated for Ph-GuT_1_-Di_1_ and Ph/Chl-GuT_1_-Di_1_ in the powder form (dried with TELSTAR freeze dryer, Cryodos, Spain) using a Fourier transform infrared (FT-IR) apparatus (Frontier, PerkinElmer, Beaconsfield, UK) equipped with an attenuated total reflectance (ATR) accessory (PerkinElmer, Beaconsfield, UK), operated by the Spectrum software (PerkinElmer, Beaconsfield, UK).

#### 3.8.2. Particle Size Distribution

Particle size distribution was evaluated using a Particle Size Analyser (Brookhaven Instruments Corporation, operated by particle sizing v.5 Brookhaven instruments software, Holtsville, NY, USA). Particle size determination was performed for Ph-GuT_1_-Di_1_ and Ph/Chl-GuT_1_-Di_1_ in solution, and polymeric layer, as well as secondary layer in powder. In what concerns to solution, the droplets of Ph-GuT_1_-Di_1_ and Ph/Chl-GuT_1_-Di_1_ were dispersed in ethanol 99% and evaluated just after preparation. For the dried particles, a small amount of powder was dispersed in ethanol 99% and sonicated (SOLTEC SONICA 2200 MH S360 Hz, Milano, Italy) for 7 min to avoid probable agglomeration and discover the inner layer. Particle’s size was characterized regarding mean size in volume and number. The operations were carried out in 6 runs of 1 min at 21 °C.

#### 3.8.3. Scanning Electron Microscopy (SEM)

The particles surface morphology was characterized by surface structural morphology under image performed by SEM (Fei Quanta 400 FEG ESEM/EDAX Pegasus X4M, The Netherlands). Dried particles were put on a brass stub (carbon stub) using a double-sided adhesive tape, dried with N_2_ and then coated with an electrical conductive (a thin layer of gold) in vacuum by sputtering in a Jeol JFC 100 apparatus. The operation was performed at Centro de Materiais da Universidade do Porto (CEMUP).

#### 3.8.4. Zeta Potential (Surface Charge)

The surface charge was measured in a ZetaPLAS (Zeta Potential Analyser, Brookhaven Instruments Corporation, operated by PALS Zeta Potential Analyser v.5 Brookhaven Instruments software, Holtsville, NY, USA). The process was evaluated in 6 runs of 30 s at 21 °C. All samples were analyzed regarding the external and inner layers.

#### 3.8.5. Molecular Docking Arrangement

The 3D structure of phospholipids and selected phenolic compounds were downloaded from the ChemSpider database. The structures were prepared and refined using Ligprep application in Maestro 12.8 (New York, Schrödinger). The 3D model of bilayers were generated using a MemGen webserver, which is specified for lipid membrane simulation systems [66]. The generated models were downloaded as PDB format and subjected for additional optimization using OPLS3 force field (Maestro 12.8). For the investigation interaction of selected phenolic compounds with the prepared bilayers, a grid box (x = 8.96, y = 28.10, z = 30.36, Size of 40, 40, 40 Å) was generated. The interaction of ligands with bilayers was carried out using the Glide application with extra precision (XP) level in Maestro 12.8. For each ligand, five poses have been used to evaluate docking interactions.

### 3.9. Statistical Analysis

The results were expressed as mean ± standard deviation (for chemical assays) or mean ± standard error of mean (for bioassays), for at least 2 independent experiments. One-way ANOVA test followed by post-hoc comparisons according to Tukey’s HSD were used to investigate significant differences between samples. Statistical differences between two groups under consideration were evaluated by Student’s *t*-test. Values were considered statistically significant if *p* < 0.05. IBM SPSS 25 for Windows (IBM Corp., Armonk, NY, USA) and the GraphPad Prism 7.0 (San Diego, CA, USA) software were used for data treatment.

## 4. Conclusions

Concluding, this study shows that the different parts of *G. tinctoria* are rich in nutrients, specifically protein and fiber, and could be an interesting alternative source of such components for human diet. *G. tinctoria* is also a source of antioxidants, namely, phenolic compounds, particularly their baby and adult leaves. Besides, it was possible to significantly enrich (more than two-fold) the plant phenolic fraction using adsorbent resins (best overall performance for Diaion^TM^ HP20LX), also showing that this enriched fraction presented anti-tumoral properties in breast and pancreas cancer cell lines. 

Regarding the nano-phytosome formulation, a highest product yield (53–70%) was obtained for cholesterol-conjugated phytosome (attributed to a considerable stability confirmed by molecular docking). Furthermore, the surface charge of all particles was found to be in an acceptable negative mood in which the nano-phytosome and cholesterol-conjugated phytosome provided the highest values which considerably enhance stability, flexibility, and permeability of the substrate to cell membrane (especially through a brain broad barrier or skin penetration), resulting in improved penetration performance.

In addition, the schematic formation of the polymeric layer was estimated based on the probable formation of the coating layer expected and in accordance with particle size distribution of inner layers. The following outcome may have an important impact on future research regarding the mechanism of drug permeability in human body. Hence, the molecular docking computational assay confirmed the accurate arrangement of phenolic compounds within the bilayer in terms of affinity based on polarity and size of phenolic compounds. Those features were confirmed by binding energy and schematic molecular arrangement images reported from molecular docking output. Although molecular docking arrangement was first developed for protein arrangement, it was shown, in the current study, that this methodology could be easily adjusted to all types of lipid-based layers for future research. 

As a final remark, this work intends to contribute to highlight the potential of *G. tinctoria* as a source of nutrients and bioactive compounds and present an innovative strategy for its valorization.

## Figures and Tables

**Table 1:** Nutritional composition of different parts of *G. tinctoria*.

	Roots	Inflorescences	Baby Leaves	Adult Leaves
Moisture	61.20 ± 0.11 ^d^	91.35 ± 0.06 ^a^	90.55 ± 0.10 ^b^	85.95 ± 0.08 ^c^
Ash	5.13 ± 0.05 ^d^	11.37 ± 0.12 ^a^	7.13 ± 0.06 ^c^	9.96 ± 0.12 ^b^
Protein	2.53 ± 0.05 ^d^	8.79 ± 0.31 ^c^	10.55 ± 0.23 ^b^	13.62 ± 0.85 ^a^
Fat	0.03 ± 0.00 ^d^	7.96 ± 0.13 ^a^	1.14 ± 0.16 ^c^	2.10 ± 0.23 ^b^
Total Fiber	12.62 ± 0.53 ^d^	41.31 ± 0.55 ^b^	36.54 ± 0.43 ^c^	44.19 ± 0.32 ^a^
Insoluble Fiber	10.42 ± 0.25 ^c^	39.24 ± 1.21 ^a^	31.55 ± 1.49 ^b^	39.94 ± 0.93 ^a^
Soluble Fiber	2.21 ± 0.78 ^b^	2.07 ± 0.66 ^b^	4.98 ± 1.06 ^a^	4.25 ± 1.26 ^a^
Available Carbohydrates	79.75 ± 0.59 ^a^	30.76 ± 0.78 ^c^	44.78 ± 0.46 ^b^	30.24 ± 1.60 ^c^

The results are expressed in g/100 g dry weight (except for moisture: g/100 g fresh weight). In each line, different small letters represent significant differences between samples (*p* < 0.05).

**Table 2:** Total Phenolics Content (TPC), Total Flavonoids Content (TFC), Ferric Reducing Antioxidant Power (FRAP), and DPPH^●^ scavenging ability (SA) of different parts of *G. tinctoria*.

	Roots	Inflorescences	Baby Leaves	Adult Leaves
TPC (mg GAE/g)	115.4 ± 3.0 ^d^	176.2 ± 3.8 ^b^	308.0 ± 2.7 ^a^	151.4 ± 13.8 ^c^
TFC (mg CE/g)	30.1 ± 1.06 ^a^	13.76 ± 0.59 ^c^	11.86 ± 0.28 ^d^	18.28 ± 0.66 ^b^
FRAP (mmol FSE/g)	5677 ± 165 ^c^	6427 ± 205 ^b^	13153 ± 214 ^a^	6437 ± 276 ^b^
DPPH^●^ SA(mg TE/g)	220.9 ± 13.3 ^c^	542.3 ± 86.1 ^b^	705.0 ± 26.1 ^a^	515.2 ± 65.9 ^b^

In within each line, different small letters represent significant differences between samples (*p* < 0.05). GAE, gallic acid equivalents; CE, catechin equivalents; FSE, ferrous sulfate equivalents; TE, Trolox equivalents; SA, scavenging ability.

**Table 3:** Results of extraction and enrichment for the different fractions.

Sample	Code	Yield	TFC (mg CE/L)	ER	TPC (mg GAE/L)	ER
Hydroethanolic Extract	GuT_1_	58.00	24.74 ± 0.43 ^c^	-	34.93 ± 1.53 ^e^	-
E-GuT of Di	GuT_1_-Di_1_	38.55	43.14 ± 0.25 ^a^	1.74	82.82 ± 4.02 ^b^	2.37
DF-GuT of Di	GuT_1_-Di_2_	DC *	01.26 ± 0.43 ^g^	0.05	3.05 ± 0.06 ^f^	0.09
E-GuT of Re	GuT_1_-Re_1_	36.11	21.26 ± 0.87 ^cd^	0.86	77.84 ± 0.76 ^c^	2.23
DF-GuT of Re	GuT_1_-Re_2_	DC *	02.42 ± 0.25 ^f^	0.10	3.01 ± 0.18 ^f^	0.10
Ultrasonication Extract	GuT_2_	15.29	09.96 ± 0.00 ^e^	-	41.84 ± 2.47 ^d^	-
E-GuT of Di	GuT_2_-Di_1_	21.00	18.51 ± 0.25 ^d^	1.86	80.38 ± 2.07 ^bc^	1.92
DF-GuT of Di	GuT_2_-Di_2_	DC *	02.57 ± 0.43 ^f^	0.26	3.29 ± 0.70 ^f^	0.10
E-GuT of Re	GuT_2_-Re_1_	22.00	37.78 ± 0.43 ^b^	3.79	87.40 ± 2.22 ^a^	2.09
DF-GuT of Re	GuT_2_-Re_2_	DC *	01.70 ± 0.43 ^g^	0.17	0.93 ± 0.34 ^g^	0.04

In each column, different small letters represent significant differences between samples (*p* < 0.05). DC, discarded; TFC, total flavonoids content; CE, catechin equivalents; TPC, total phenolics content; GAE, gallic acid equivalents; ER, Enrichment ratio. E-GuT; Enriched fraction of GuT. DF-GuT; Discarded fraction of GuT. * Discarded fractions have undesirable compounds such as sugars and chlorophylls.

**Table 4:** Particle size distribution regarding the effective diameter in number and volume as well as zeta potential of inner and external layer.

	Sample Name	Yield	ED ^1^ In Volume	ED ^1^ In Number	ZP ^1^ Inner Layer	ZP ^1^ External Layer
Lipid layer	Ph-GuT_1_-Di_1_	-	120.0	33.5	−67.50 ± 1.43	
	Ph/Chl-GuT_1_-Di_1_	-	354.4	140.2	−62.57 ± 2.95	
Polymeric layer	GuT_1_-Di_1_-PE	34.55	1196.5	916.5	−29.72 ± 1.32	−16.21 ± 8.44
	GuT_1_-Di_1_-Malto	46.00	397.9	327.1	−33.82 ± 0.76	−31.40 ± 10.29
	GuT_1_-Di_1_-Stch	68.55	509.1	355.0	−55.01 ± 3.24	−53.47 ± 1.94
	GuT_1_-Di_1_-PE/Stch	38.00	824.6	737.2	−43.07 ± 2.17	−28.24 ± 3.06
Secondary layer	Ph-GuT_1_-Di_1_-PE	43.54	1364.6	1016.1	−21.26 ± 7.19	−17.21 ± 8.34
	Ph-GuT_1_-Di_1_-Malto	37.23	411.5	226.3	−33.82 ± 0.76	−39.14 ± 5.92
	Ph-GuT_1_-Di_1_-Stch	47.38	523.1	446.7	−31.38 ± 3.95	−25.37 ± 1.75
	Ph-GuT_1_-Di_1_-PE/Stch	41.67	951.2	752.7	−44.76 ± 3.38	−18.87 ± 9.20
Secondary layer	Ph/Chl-GuT_1_-Di_1_-PE	68.74	1207.9	1073.0	−31.67 ± 1.69	−14.99 ± 2.79
	Ph/Chl-GuT_1_-Di_1_-Malto	66.22	392.7	212.7	−34.18 ± 3.41	−29.31 ± 3.38
	Ph/Chl-GuT_1_-Di_1_-Stch	70.22	680.5	555.1	−43.07 ± 2.17	−38.46 ± 1.94
	Ph/Chl-GuT_1_-Di_1_-PE/Stch	52.97	707.6	528.1	−34.40 ± 2.84	−30.09 ± 1.99

^1^ ED; Effective Diameter, ZP; Zeta Potential. The effective diameter was reported on nm, and zeta potential was reported on mV.

**Table 5:** Binding energy of docked molecule expressed on Kcal/mol.

	Phenolic Structure	Docking Score			Grid Energy		
		Min	Max	Average	Min	Max	Average
Phytosome designed with lecithin	Gallic acid	−4.47	−4.86	−4.73 ± 0.23	−29.98	−31.37	−30.83 ± 0.75
	Ellagic acid	−4.32	−4.47	−4.31 ± 0.15	−31.33	−4.47	−32.88 ± 1.31
	Quercetin	−5.31	−5.41	−5.05 ± 0.50	−29.90	−34.23	−33.04 ± 2.57
	Catechin	−3.50	−5.36	−4.49 ± 0.66	−19.71	−38.93	−29.14 ± 9.18
	Epicatechin	−3.38	−5.23	−4.36 ± 0.66	−22.43	−39.96	−33.05 ± 6.71
Phytosome designed with lecithin conjugated with cholesterol	Gallic acid	−4.06	−4.13	−4.09 ± 0.04	−12.66	−15.75	−14.62 ±1.70
	Ellagic acid	−3.54	−3.75	−3.62 ± 0.10	−18.79	−19.24	−19.05 ± 0.21
	Quercetin	−5.12	−6.79	−5.69 ± 0.65	−23.47	−26.29	−25.01 ± 1.15
	Catechin	−5.93	−6.95	−6.42 ± 0.44	−21.62	−25.47	−23.51 ± 1.41
	Epicatechin	−4.89	−7.15	−6.50 ± 0.57	−20.84	−25.09	−24.09 ± 1.49

Docking score exhibited the number of hydrogen bindings occurring between structures. Lower docking scores denote a higher hydrogen binding.

## Data Availability

The data presented in this study are available on request from the corresponding author.

## Sample Availability

Samples of the compounds are not available from the authors.

## References

[1] Bernardini S., Tiezzi A., Laghezza M.V., Ovidi E. (2018). Natural products for human health: An historical overview of the drug discovery approaches. Nat. Prod. Res..

[2] Williams P.A., Ogle C.C., Timmins S.M., La C.G.D., Clarkson J. (2005). Chilean Rhubarb (Gunnera Tinctoria): Biology, Ecology and Conservation Impacts in New Zealand.

[3] Fuller D.Q., Hickey L.J. (2005). Systematics and leaf architecture of the Gunneraceae. Bot. Rev..

[4] Gioria M., Osborne B.A. (2013). Biological flora of the british Isles: *Gunnera tinctoria*. Ecology.

[5] Silva L., Tavares J., Pena A. Ecological basis for the control of *Gunnera tinctoria* in São Miguel Island. Proceedings of the Second International Weed Control Congress Copenhagen.

[6] Petzold G., Catril G., Duarte C. (2006). Caracterización fisicoquímica de pecíolos del pangue (*Gunnera tinctoria*). Rev. Chil. Nutr..

[7] Estomba D., Ladio A., Lozada M. (2006). Medicinal wild plant knowledge and gathering patterns in a Mapuche community from North-western Patagonia. J. Ethnopharmacol..

[8] Sabando C., Rodríguez-Díaz M., Ide W., Pastene E., Avello M., Simirgiotis M., Rojas S., Villarroel E., Silva-Grecchi T., Gutiérrez C. (2020). Improvement of endothelial function by *Gunnera tinctoria* extract with antioxidant properties. J. Biol. Res..

[9] Silva C., Correia-Branco A., Andrade N., Ferreira A.C., Soares M.L., Sonveaux P., Stephenne J., Martel F. (2019). Selective pro-apoptotic and antimigratory effects of polyphenol complex catechin: Lysine 1: 2 in breast, pancreatic and colorectal cancer cell lines. Eur. J. Pharmacol..

[10] Karakaya S. (2004). Bioavailability of phenolic compounds. Crit. Rev. Food Sci. Nutr..

[11] Grgić J., Šelo G., Planinić M., Tišma M., Bucić-Kojić A. (2020). Role of the encapsulation in bioavailability of phenolic compounds. Antioxidants.

[12] Bayati M., Mehrbod P., Ebrahimi S.N. (2020). Natural products as inhibitors of COVID-19 main protease–A virtual screening by molecular docking. Res. Square.

[13] Ballester P.J., Mitchell J.B. (2010). A machine learning approach to predicting protein–ligand binding affinity with applications to molecular docking. J. Bioinform..

[14] Commission E. (2006). Commission Directive 2006/141/EC of 22 December 2006 on infant formulae and follow-on formulae and amending Directive 1999/21/EC. Off. J. Eur. Union.

[15] Zamorano P., Rojano B.I., Morales M., Magariños H., Godoy P., Muñoz O. (2017). Biological and antioxidant activity of *Gunnera tinctoria* (Nalca). J. Med. Plants Res..

[16] Davoodi M., Rustaiyan A., Ebrahimi S.N. (2018). Monoterpene flavonoid from aerial parts of *Satureja khuzistanica*. Rec. Nat. Prod..

[17] Kokabi M., Ebrahimi S.N. (2020). Polyphenol rnriched extract of pomegranate peel; A novel precursor for the biosynthesis of zinc oxide nanoparticles and application in sunscreens. Pharm. Sci..

[18] Scordino M., Di Mauro A., Passerini A., Maccarone E. (2005). Selective recovery of anthocyanins and hydroxycinnamates from a byproduct of citrus processing. J. Agric. Food Chem..

[19] Mahato N., Sinha M., Sharma K., Koteswararao R., Cho M.H. (2019). Modern extraction and purification techniques for obtaining high purity food-grade bioactive compounds and value-added co-products from citrus wastes. Foods.

[20] Bridi R., Giordano A., Peñailillo M.F., Montenegro G. (2019). Antioxidant effect of extracts from native Chilean plants on the lipoperoxidation and protein oxidation of bovine muscle. Molecules.

[21] Hussain T., Tan B., Yin Y., Blachier F., Tossou M.C., Rahu N. (2016). Oxidative stress and inflammation: What polyphenols can do for us?. Oxid. Med. Cell. Longev..

[22] Yilmaz Y., Toledo R.T. (2004). Major flavonoids in grape seeds and skins: Antioxidant capacity of catechin, epicatechin, and gallic acid. J. Agric. Food Chem..

[23] Oyenihi A., Smith C. (2019). Are polyphenol antioxidants at the root of medicinal plant anti-cancer success?. J. Ethnopharmacol..

[24] Teleki A., Hitzfeld A., Eggersdorfer M. (2013). 100 years of vitamins: The science of formulation is the key to functionality. Kona.

[25] Tontul I., Topuz A. (2017). Spray-drying of fruit and vegetable juices: Effect of drying conditions on the product yield and physical properties. Trends Food Sci. Technol..

[26] Cano-Higuita D.M., Vélez H.A.V., Telis V.R.N. (2015). Microencapsulation of turmeric oleoresin in binary and ternary blends of gum Arabic, maltodextrin and modified starch. Cienc. Agrotecnol..

[27] Jovanović A.A., Lević S.M., Pavlovic V.B., Markovic S.B., Pjanovic R.V., Đorđević V.B., Nedović V., Bugarski B.M. (2021). Freeze versus spray drying for dry wild thyme (*Thymus serpyllum* L.) extract formulations: The impact of gelatin as a coating material. Molecules.

[28] Keshani S., Daud W.R.W., Nourouzi M., Namvar F., Ghasemi M. (2015). Spray drying: An overview on wall deposition, process and modeling. J. Food Eng..

[29] Wang Y., Xie Y., Xu D., Lin X., Feng Y., Hong Y. (2014). Hydroxypropyl methylcellulose reduces particle adhesion and improves recovery of herbal extracts during spray drying of Chinese herbal medicines. Dry. Technol..

[30] Hou Z., Li Y., Huang Y., Zhou C., Lin J., Wang Y., Cui F., Zhou S., Jia M., Ye S. (2013). Phytosomes loaded with mitomycin C–soybean phosphatidylcholine complex developed for drug delivery. Mol. Pharm..

[31] Zhang J., Tang Q., Xu X., Li N. (2013). Development and evaluation of a novel phytosome-loaded chitosan microsphere system for curcumin delivery. Int. J. Pharm..

[32] Nazari M., Ghanbarzadeh B., Kafil H.S., Zeinali M., Hamishehkar H. (2019). Garlic essential oil nanophytosomes as a natural food preservative: Its application in yogurt as food model. Colloids Interface Sci. Commun..

[33] Souza J.E.D., Casanova L.M., Costa S.S. (2015). Bioavailability of phenolic compounds: A major challenge for drug development?. Rev. Fitos (Rio J.).

[34] Khan I., Saeed K., Khan I. (2019). Nanoparticles: Properties, applications and toxicities. Arab. J. Chem..

[35] Rasaee S., Ghanbarzadeh S., Mohammadi M., Hamishehkar H. (2014). Nano phytosomes of quercetin: A promising formulation for fortification of food products with antioxidants. J. Pharm. Sci..

[36] Pieczykolan E., Kurek M.A. (2019). Use of guar gum, gum arabic, pectin, beta-glucan and inulin for microencapsulation of anthocyanins from chokeberry. Int. J. Biol..

[37] Ansari F., Sobhani A., Salavati-Niasari M. (2018). Simple sol-gel synthesis and characterization of new CoTiO_3_/CoFe_2_O_4_ nanocomposite by using liquid glucose, maltose and starch as fuel, capping and reducing agents. J. Colloid Interface Sci..

[38] Papoutsis K., Golding J.B., Vuong Q., Pristijono P., Stathopoulos C.E., Scarlett C.J., Bowyer M. (2018). Encapsulation of citrus by-product extracts by spray-drying and freeze-drying using combinations of maltodextrin with soybean protein and ι-Carrageenan. Foods.

[39] Cortes U.A.B., Gutiérrez M.C., Mendoza D.G., Salitre L.G., Vargas A.S., Catzim C.E.A., Durán C.C., Valenzuela B.E.L. (2021). Microencapsulation and antimicrobial activity of extract acetone-methanol of *Hibiscus sabdariffa* L. using a blend modified starch and pectin as a wall material. Ind. Crops Prod..

[40] Clogston J.D., Patri A.K., McNeil S. (2011). Zeta potential measurement. Characterization of Nanoparticles Intended for Drug Delivery.

[41] Campos J.C., Ferreira D.C., Lima S., Reis S., Costa P.J. (2019). Swellable polymeric particles for the local delivery of budesonide in oral mucositis. Int. J. Pharm..

[42] Singh R.P., Gangadharappa H., Mruthunjaya K. (2018). Phytosome complexed with chitosan for gingerol delivery in the treatment of respiratory infection: In vitro and in vivo evaluation. Eur. J. Pharm. Sci..

[43] Hindarto C.K., Surini S., Saputri F.C., Irawan C. (2017). In vivo evaluation of luteolin-loaded phytosome. J. Pharm. Innov..

[44] Nagaraju P.G., Sindhu P., Dubey T., Chinnathambi S., Priyadarshini P.C.G., Rao P.J. (2021). Influence of sodium caseinate, maltodextrin, pectin and their Maillard conjugate on the stability, in vitro release, anti-oxidant property and cell viability of eugenol-olive oil nanoemulsions. Int. J. Biol. Macromol..

[45] Jeong O., Shin M. (2018). Preparation and stability of resistant starch nanoparticles, using acid hydrolysis and cross-linking of waxy rice starch. Food Chem..

[46] Li X., Kong X., Shi S., Zheng X., Guo G., Wei Y., Qian Z. (2008). Preparation of alginate coated chitosan microparticles for vaccine delivery. BMC Biotechnol..

[47] Choudhary M.I., Shaikh M., tul-Wahab A., ur-Rahman A. (2020). In silico identification of potential inhibitors of key SARS-CoV-2 3CL hydrolase (Mpro) via molecular docking, MMGBSA predictive binding energy calculations, and molecular dynamics simulation. PLoS ONE.

[48] Bozzuto G., Molinari A. (2015). Liposomes as nanomedical devices. Int. J. Nanomed..

[49] Ghanbarzadeh B., Babazadeh A., Hamishehkar H. (2016). Nano-phytosome as a potential food-grade delivery system. Food Biosci..

[50] AOAC (2012). Official Methods of Analysis of Associationo Of Analytical Chemistry.

[51] Ayadi M., Abdelmaksoud W., Ennouri M., Attia H. (2009). Cladodes from Opuntia ficus indica as a source of dietary fiber: Effect on dough characteristics and cake making. Ind. Crops Prod..

[52] Food and Agriculture Organization (2003). Food energy—Methods of analysis and conversion factors. Report of a technical workshop. Food and Agriculture Organization of the United Nations Technical Workshop Report 77.

[53] Costa A.S., Alves R.C., Vinha A.F., Barreira S.V., Nunes M.A., Cunha L.M., Oliveira M.B.P. (2014). Optimization of antioxidants extraction from coffee silverskin, a roasting by-product, having in view a sustainable process. Ind. Crops Prod..

[54] Puga H., Alves R.C., Costa A.S., Vinha A.F., Oliveira M.B.P. (2017). Multi-frequency multimode modulated technology as a clean, fast, and sustainable process to recover antioxidants from a coffee by-product. J. Clean. Prod..

[55] Shehzad O., Jin-Ha I., Park Y., Wan-Ha Y., Shik-Kim Y. (2011). Development of a rapid and convenient method to separate eight ginsenosides from Panax ginseng by high-speed counter-current chromatography coupled with evaporative light scattering detection. J. Sep. Sci..

[56] Silva C., Nunes C., Correia-Branco A., Araújo J.R., Martel F. (2017). Insulin exhibits an antiproliferative and hypertrophic effect in first trimester human extravillous trophoblasts. Reprod. Sci..

[57] Andrade N., Araújo J.R., Correia-Branco A., Carletti J.V., Martel F. (2017). Effect of dietary polyphenols on fructose uptake by human intestinal epithelial (Caco-2) cells. J. Funct. Foods.

[58] Azevedo C., Correia-Branco A., Araújo J.R., Guimaraes J.T., Keating E., Martel F. (2015). The chemopreventive effect of the dietary compound kaempferol on the MCF-7 human breast cancer cell line is dependent on inhibition of glucose cellular uptake. Nutr. Cancer.

[59] Bradford M.M. (1976). A rapid and sensitive method for the quantitation of microgram quantities of protein utilizing the principle of protein-dye binding. Anal. Biochem..

[60] Fathi F., Ebrahimi S.N. (2021). Investigation of physiochemical properties nanophytosome obtained from of polyphenolic enrich fraction of *Satureja khuzistanica* by freeze-drying. Nashrieh Shimi va Mohandesi Shimi Iran.

[61] Gandhi A., Dutta A., Pal A., Bakshi P. (2012). Recent trends of phytosomes for delivering herbal extract with improved bioavailability. J. Pharmacogn. Phytochem..

[62] Anwar E., Farhana N. (2018). Formulation and evaluation of phytosome-loaded maltodextrin-gum Arabic microsphere system for delivery of *Camellia sinensis* extract. J. Young Pharm..

[63] El-Messery T.M., Altuntas U., Altin G., Özçelik B. (2020). The effect of spray-drying and freeze-drying on encapsulation efficiency, in vitro bioaccessibility and oxidative stability of krill oil nanoemulsion system. Food Hydrocoll..

[64] Tuyen C.K., Nguyen M.H., Roach P.D., Stathopoulos C.E. (2014). Microencapsulation of gac oil: Optimisation of spray drying conditions using response surface methodology. J. Powder Technol..

[65] Santana A.A., de Oliveira R.A., Kurozawa L.E., Park K.J. (2014). Microencapsulation of pequi pulp by spray drying: Use of modified starches as encapsulating agent. Eng. Agric..

[66] Knight C.J., Hub J.S. (2015). MemGen: A general web server for the setup of lipid membrane simulation systems. J. Bioinform..

